# Does simulated walking cause gapping of meniscal repairs?

**DOI:** 10.1186/s40634-016-0047-3

**Published:** 2016-03-15

**Authors:** Patrick C. McCulloch, Hugh L. Jones, Kendall Hamilton, Michael G. Hogen, Jonathan E. Gold, Philip C. Noble

**Affiliations:** Methodist Center for Sports Medicine, Houston Methodist Hospital, Houston, TX USA; Institute of Orthopedic Research & Education, Houston, TX USA; Spectrum Health Medical Group, Grand Rapids, MI USA

## Abstract

**Background:**

The objective of rehabilitation following meniscal repair is to promote healing by limiting stresses on repairs, while simultaneously preserving muscle strength and joint motion. Both protective protocols limiting weight bearing and accelerated which do not, have shown clinical success. This study assesses the effects of physiologic gait loading on the kinematic behavior of a repaired medial meniscus.

**Methods:**

The medial menisci of eight fresh cadaveric knees were implanted with arrays of six 0.8–1.0 mm beads. Pneumatic actuators delivered muscle loads and forces on the knee as each specimen was subjected to a simulated stance phase of gait. Meniscus motion was measured at loading response, mid stance, and toe-off positions. Measurements were performed using biplanar radiography and RSA, with each knee: (a) intact, (b) with posterior longitudinal tear, and (c) after inside-out repair.

**Results:**

The tissue spanning the site of the longitudinal tear underwent compression rather than gapping open in all states (intact [I], torn [T] and repaired [R] states). Average compression at three sites along the posterior half of the meniscus was: posterior horn −0.20 ± 0.08 mm [I], −0.39 ± 0.10 mm [T], and −0.20 ± 0.06 mm [R] (*p* = 0.15); junction of posterior horn and body −0.11 ± 0.12 mm [I], −0.21 ± 12 mm [T], −0.17 ± 0.09 mm [R] (*p* = 0.87); and adjacent to the medial collateral ligament −0.07 ± 0.06 mm [I], −0.29 ± 0.13 mm [T], −0.07 ± 0.17 mm [R] (*p* = 0.35). The entire meniscus translated posteriorly from mid-stance to toe off. Displacement was greatest in the torn state compared to intact, but was not restored to normal levels after repair.

**Conclusion:**

The edges of a repaired longitudinal medial meniscal tear undergo compression, not gapping, during simulated gait.

## Background

Meniscal repair is a common procedure in active patients with healthy articular cartilage (Paxton et al. 2011; Stein et al. 2010). Meniscal repairs must provide mechanical stability under joint load and motion until the meniscus heals. However, not all repaired menisci heal, with published failure rates ranging from 16 to 21 % in the literature (Barber 1994; Morgan and Casscells 1986; Morgan et al. 1991; Richards et al. 2005). Surgeons have often chosen to limit range of motion and/or weight bearing to protect the repair early in the healing process. However, there is no published link between allowing normal gait and clinical failure (Barber 1994; McLaughlin et al. 1994; Shelbourne and Nitz 1990; Stärke et al. 2009).

The objective of rehabilitation following meniscal repair is to promote healing by limiting stresses on repairs, while simultaneously preserving muscle strength and joint motion. Both protective protocols which limit weight bearing, as well as accelerated protocols which do not, have shown clinical success (Barber 1994; Mariani et al. 1996; McLaughlin et al. 1994). Protective protocols advocate non-weight bearing and motion from 0 to 90° for three to six weeks and no deep flexion weight bearing for three months. Others allow weight bearing, but only with the knee locked in extension rather than normal gait. This can have deleterious effects on cartilage, muscle and knee motion, especially in patients who undergo concomitant ligamentous procedures (Barber 1994; Brantigan and Voshell 1941; Ganley et al. 2000; Klein et al. 1982; Lin et al. 2013; Mariani et al. 1996; McLaughlin et al. 1994; Richards et al. 2005; Schimmer et al. 1998; Shelbourne and Nitz 1990; Stärke et al. 2009). Additionally, prolonged immobilization and a delay in return to play can lead athletes to prefer meniscectomy, despite evidence for less radiographic degeneration and higher return to pre-injury activity level associated with repair (Barber 1994; Dowdy et al. 1995; Mariani et al. 1996; Schimmer et al. 1998). The belief that peripheral meniscal tears undergo edge separation at increasing degrees of flexion was the major impetus for protective protocols (Ganley et al. 2000; McLaughlin et al. 1994; Morgan and Casscells 1986). However, our previous work using meniscal RSA demonstrated that high flexion angles resulted in compression across posterior tear edges, rather than gapping (Lin et al. 2013). This study did not address the effect of weight-bearing across the tear, which became the focus of this investigation.

Clinical studies examining the effect of partial weight bearing on meniscal healing have suggested that functional loading may promote collagen formation after repair, without tear separation or early failure (Ganley et al. 2000; Taylor et al. 2004). The lack of clinical evidence supporting the use of an extension brace or protected weight bearing with crutches following meniscal injury has stimulated our interest in the kinematic behavior of meniscal repair during walking. This study was performed to answer the following questions:

1. How does the medial meniscus move during gait, and what pattern of internal deformation occurs within the meniscus itself?
2. Under the joint loads seen in gait, how do meniscal displacement and deformation change in the presence of a longitudinal tear? Specifically, does gait loading cause transverse or vertical separation of longitudinal tears?
3. Is the displacement and deformation of the native meniscus restored after suture repair of a longitudinal tear?

Answers to these questions would help to define if physiologic loading causes harmful separation of meniscus repairs that would limit the use of accelerated rehabilitation. We hypothesize that joint reactive forces produced during stance phase will not cause significant gapping along the torn edges of the repair construct. It is also our hypothesis that the repaired meniscus will exhibit similar biomechanical behavior to the intact meniscus in response to load.

## Methods

Eight fresh-frozen human lower limbs, ranging in age from 25 to 64 (mean age of 50), were selected for study inclusion after a clinical and arthroscopic evaluation was performed to confirm the absence of ligamentous, capsular, or meniscal injuries. Each specimen was resected 20 cm from the joint line both proximally and distally leaving the quadriceps, hamstrings, capsule, and ligamentous structures intact. The femur and tibia of each specimen were then potted in casting resin (Smooth-Cast 300) and PVC piping 10 cm from the joint line. To allow for independent loading when attached to the testing apparatus, the hamstring muscles were grouped together, as were the individual quadriceps muscles. Number two ETHIBOND EXCEL® braided sutures (Ethicon, Somerville, NJ, USA) were then stitched into the tendon origins of each muscle group creating an attachment lead for loading.

**Fig. 1:**
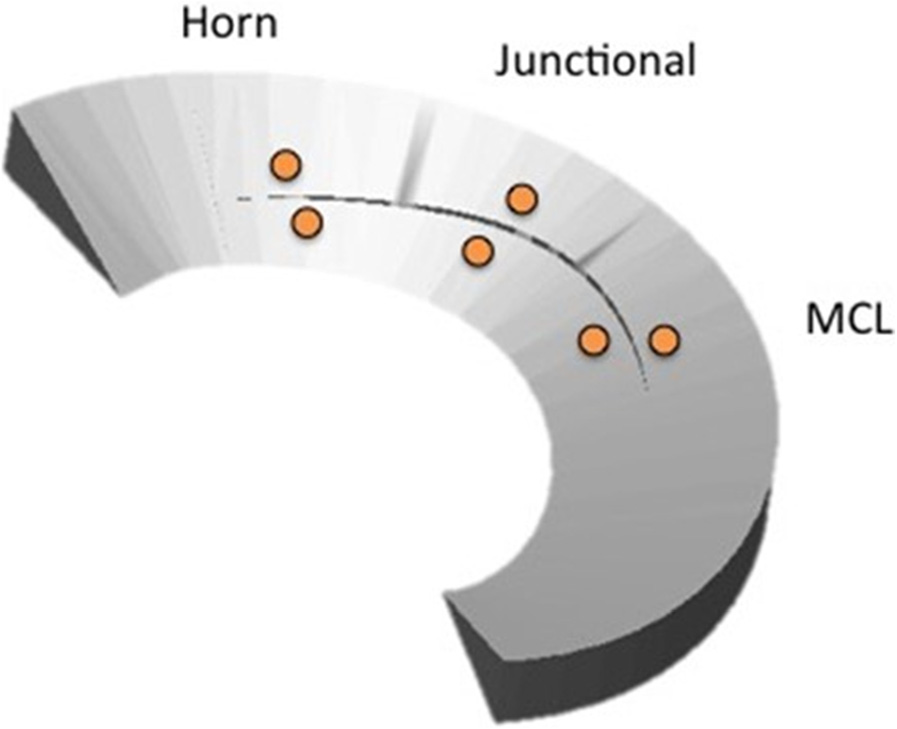
Schematic drawing Representing Bead Placement in the Medial Meniscus

**Fig. 2:**
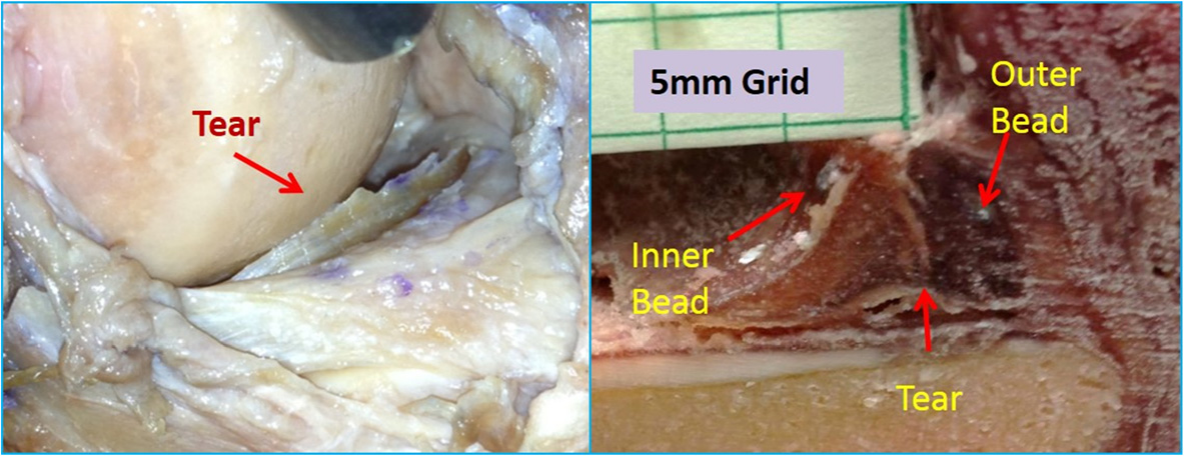
Surgical Photograph and meniscal frozen cross section

Meniscal tear creation, inside-out meniscal repair, and Roentgen Stereophotogrammetric Analysis (RSA) measurement was performed using previously published methods (Komistek et al. 1998; Li et al. 2014; Morgan and Casscells 1986). Briefly, a standard posteromedial approach was used to expose the posterior medial capsule. A capsulotomy was made longitudinally, just posterior to the medial collateral ligament (MCL) to expose the posterior aspect of the medial meniscus. Six spherical radiographic markers were implanted into the medial meniscus to allow subsequent tracking of meniscal movement using RSA. The markers were implanted as radial couples using three unique combinations of marker diameter and radiographic density (0.8 mm tantalum, 1.0 mm tantalum, and 1.0 mm stainless steel), to allow differentiation when analyzing the radiographic images. The outer meniscal markers were implanted within the red zone of the meniscus and the corresponding inner markers within the red-white zone. Custom syringe needles, guides, and seating rods were used to pierce the meniscus and fix the markers at a depth of 1 mm below the meniscal surface with cyanoacrylate. Placement of the marker couples began 10 mm from the posterior root and was evenly spaced circumferentially until MCL insertion. The average radial distance between markers within each couple was 5.3 mm, with an average circumferential spacing of 7.1 mm (Figs. 1 and 2). Two markers were also implanted in the femoral epicondyles for verification of tibio-femoral kinematics during testing. Four additional markers of 3 mm in diameter were fixed within the cortical bone of the tibia metaphysis to serve as fiduciaries for registration of later described computer kinematic models. Following placement of the beads, the capsulotomy was closed with interrupted #0 VICRYL™ suture (Ethicon, Somerville, NJ, USA).

**Fig. 3:**
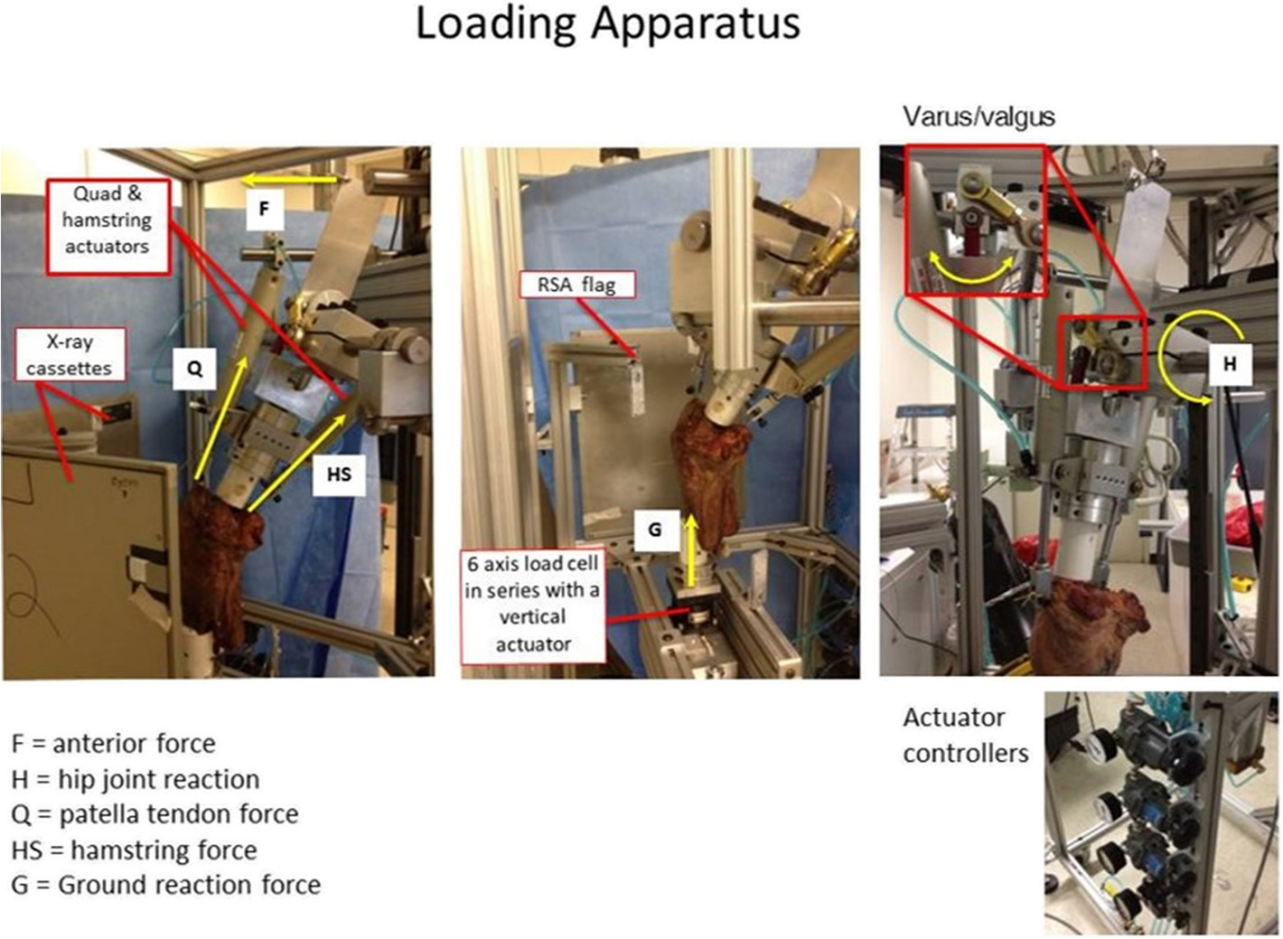
Photographs of the Custom loading apparatus used to load each knee specimen and create the required joint reactive forces during simulated weight bearing

Upon mechanical testing, each specimen was mounted upright to a custom loading apparatus. The tibia was oriented with its longitudinal axis aligned perpendicular to the floor, while the femur was attached to a loading head that allowed translational and rotational freedom in all cardinal planes during knee motion (Bylski-Austrow et al. 1994; Komistek et al. 1998; Pujol et al. 2008). Once positioned, the muscle leads were separately attached to pneumatic actuators mounted on the loading head such that their lines of action were parallel to the femoral canal (Rankin et al. 2002). A third pneumatic actuator, which applied reported ground reaction forces of normal gait, was attached to the tibial potting clamp in series with a six axis load cell (Fig. 3).

During testing, the knees were cycled 10 times through a range of motion simulating gait and measurements were taken at three instants during stance phase: Heel strike loading response (15 % of gait cycle), mid-stance (40 %), and toe-off (60 %). Initially, each knee was placed in full extension and a load of 222 N (22.6 kg) was applied to the quadriceps tendon, while a load of 53 N (5.4 kg) was applied to the hamstrings. The knee was then flexed 15° into the position of loading response (contralateral toe-off). Once in position, a segmental link free body diagram of the testing configuration was used to prescribe the load settings of the three actuators necessary to induce reported physiologic joint reaction forces (Table 1) (Bylski-Austrow et al. 1994; Komistek et al. 1998; Lin et al. 2013). After these loads were applied, forces acting at the distal tibia were recorded with the mentioned six axis load cell and compared to the expected result of the free body diagram. Slight adjustments of the actuator settings were then made to dial in the expected loading result. Bi-planar radiographs were taken and the knee was further flexed into the positions of mid stance and toe-off where the loading process was repeated.

**Table 1:** Knee flexion angle and induced joint reaction forces are depicted for each gait position

Position	Description	Flexion (deg)	Joint reaction force (BW)
LR = Loading Response	Contralateral Toe-off	15	1.9 vertical 0.25 shear
MS = Mid stance	Ipsilateral Foot Flat	10	1.4 vertical 0.25 shear
TO = Toe Off	Contralateral heel strike	32	2.2 vertical 0.25 shear

Body weight was 713 N (73 kg)

*Description of Terms*: *LR* Loading Response at heel strike, *MS* Mid stance during ipsilateral foot flat position, *TO* The position of contralateral limb heel strike until the ipsilateral limb toe off position

After testing the intact state, the capsulotomy was re-opened and a longitudinal tear was created within the red-red zone using a scalpel. The tear was initiated between the bead couples in the posterior meniscus extending from just medial to the root, to the posterior edge of the MCL fibers (average length 14.8 mm). The experimental meniscal tear was repaired using a vertical mattress inside-out technique with three 2–0 ORTHOCORD® sutures (DePuy, Mitek Inc., Raynham, MA, USA). Sutures were placed on both the superior and inferior surfaces of the tear to achieve an anatomic reduction. After testing of each knee with a repaired meniscus, all sutures were removed and the testing was repeated to characterize the kinematics of acute meniscal injury.

In each of the three loading positions, the three-dimensional (3D) location of each implanted marker couple was measured, with respect to the bony surface of the tibia, using RSA. As this methodology has been well-described in literature, the technique is described only briefly here (Komistek et al. 1998; Li et al. 2014). Two film cassettes were mounted to the testing frame at 90° apart, and x-ray tubes were positioned normal to each cassette center at a distance of 130 cm. After bi-planar exposures were taken, each film was digitized with a Microtek Medi-6000 scanner (Microtek Lab Inc, Santa Fe Springs, CA, USA) at a resolution of 600 dpi. The digital images were imported into ImageJ software (NIH, Bethesda, Maryland, USA), where a threshold routine was used to automatically determine the 2D coordinates of the marker centers. This allowed calculation of the 3D location of each fiduciary and meniscal marker using custom software, with an accuracy of better than 80 microns. The 3D marker coordinates were then imported into modeling software (RapidForm, 3D Systems Rock Hill, SC, USA), where each flexion angle was overlaid via registration of the tibial fiduciaries. These gait models were used to digitally measure the marker displacements from one position relative to another. The change in marker couple separation and regional translation were averaged and compared for each specimen state. The relative motions of the beads were reported in horizontal, vertical, and 3D scalar distances. An increase in separation between the bead pairs was defined as gapping, and a decrease in couple distance was defined as compression. The position of the fiduciaries implanted within the femur and tibia were used to establish the Cartesian coordinate system that defined meniscal bead motion. All statistical comparisons between the intact, torn and repaired states were calculated using StatPlus v5 software (AnalystSoft Inc., Alexandria, VA, USA), within which a one-way analysis of variance (ANOVA) was performed. A post hoc Fisher’s Least Significance Difference procedure was employed for all findings, with a statistical significance level set at *p* < 0.05.

## Results

The translation of the meniscus in anterior-posterior (AP), medial-lateral (ML), and superior-inferior (SI) directions during simulated gait is shown in Table 2. The internal deformations of the meniscus including the bead separation/compression in the radial, vertical, and 3D planes are shown in Table 3.

**Table 2:** Translation of the Meniscus

Meniscal Translation						
	ML Displacement (mm)		AP Displacement (mm)		SI Displacement (mmm)	
State	LR to MS	MS to TO	LR to MS	MS to TO	LR to MS	MS to TO
Intact	−0.02 ± 0.01	0.02 ± 0.05	−0.63 ± 0.03	1.67 ± 0.11	−0.11 ± 0.02	−0.2 ± 0.06
Torn	−0.03 ± 0.01	−0.16 ± 0.08	−0.86 ± 0.04	3.44 ± 0.17	−0.07 ± 0.03	−0.4 ± 0.06
Repaired	−0.07 ± 0.03	0.11 ± 0.06	−0.97 ± 0.03	2.9 ± 0.14	0.14 ± 0.04	−0.27 ± 0.09

This depicts the average values for the displacement of the meniscus during gait. For medial-lateral (ML) translation, positive values indicate translation toward the MCL. For anterior-posterior (AP), positive values indicate posterior translation. For superior-inferior displacement (SI), positive values indicate translation of the meniscus towards the femoral condyle

**Table 3:** Radial Separation distance between coupled bead pairs

Internal Meniscal Deformation						
	Intact		Torn		Repaired	
3D	LR to MS	MS to TO	LR to MS	MS to TO	LR to MS	MS to TO
MCL	0.04 ± 0.06	−0.07 ± 0.06	0.04 ± 0.08	−0.29 ± 0.13	−0.08 ± 0.11	−0.07 ± 0.17
Junctional	−0.17 ± 0.12	−0.11 ± 0.12	−0.06 ± 0.04	−0.21 ± 0.12	−0.13 ± 0.11	−0.17 ± 0.09
Horn	−0.13 ± 0.08	−0.2 ± 0.08	−0.07 ± 0.08	−0.39 ± 0.01	−0.01 ± 0.05	−0.2 ± 0.06
Radial						
MCL	0.02 ± 0.05	−0.11 ± 0.05	−0.02 ± 0.05	−0.23 ± 0.10	−0.14 ± 0.12	−0.04 ± 0.13
Junctional	−0.15 ± 0.1	−0.25 ± 0.10	−0.04 ± 0.03	−0.26 ± 0.08	−0.17 ± 0.14	−0.13 ± 0.01
Horn	−0.14 ± 0.8	−0.2 ± 0.08	−0.07 ± 0.07	−0.39 ± 0.08	−0.04 ± 0.06	−0.14 ± 0.1
Superior/Inferior						
MCL	0.05 ± 0.05	0.09 ± 0.05	0.12 ± 0.11	−0.21 ± 0.13	0.18 ± 0.05	−0.11 ± 0.15
Junctional	−0.08 ± 0.05	0.27 ± 0.05	−0.06 ± 0.8	0.06 ± 0.11	0.12 ± 0.09	−0.16 ± 0.07
Horn	0.01 ± 0.07	−0.05 ± 0.07	−0.05 ± 0.09	−0.12 ± 0.13	0.16 ± 0.1	−0.23 ± 0.11

3D bead deformation represents average values of the separation distance between bead couples for the combined transverse and vertical displacements in the intact, torn, and repaired meniscus. Positive values indicate the bead pairs are moving away from each other or separating. Negative values depict compression or that the bead pairs are moving closer together. Radial shows the spread difference in the horizontal plane only. The Superior/Inferior distance corresponds to the separation or compression of bead pairs in the vertical plane

**Fig. 4:**
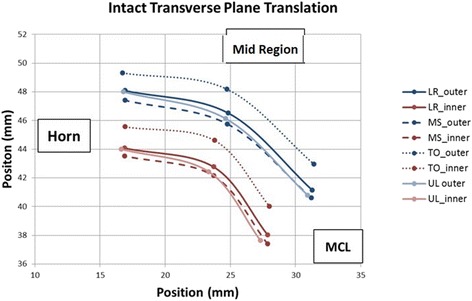
Intact Meniscus Graphic showing the combined translations and relative positions of the beads (deformations) in the transverse plane. Inner and Outer edge displacements for each state are labeled

**Fig. 5:**
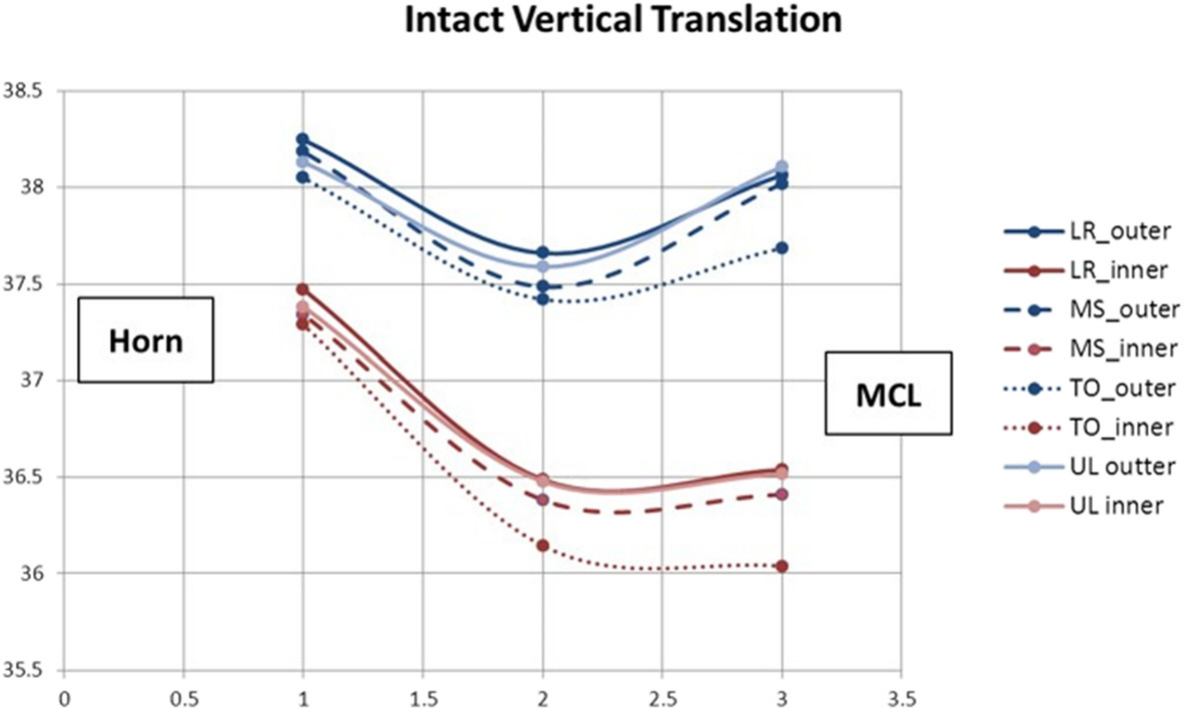
Intact Meniscus Graphic showing the combined translations and relative positions of the beads (deformations) in the vertical plane. Inner and Outer edge displacements for each state are labeled

In the intact state, the medial meniscus translated in both the anterior and posterior directions, with combined internal constrictive and bulging deformations depending on which part of the stance phase it was experiencing. During the transition from full weight bearing heel strike to mid-stance, translation of the posterior meniscus across the tibia averaged 0.63 ± 0.03 mm in the anterior direction, while displacements in the ML and SI directions were minimal. Subsequent transition into toe-off position resulted in the MCL region translating posteriorly on the tibia the most (1.89 ± 0.43 mm), followed by the junctional region (1.76 ± 0.44 mm) and further internal consolidation at all locations. As the meniscus elongated, Poisson effects generated further internal consolidation. The combined translation and deformation patterns for the intact state are shown graphically in Figs. 4 and 5.

**Fig. 6:**
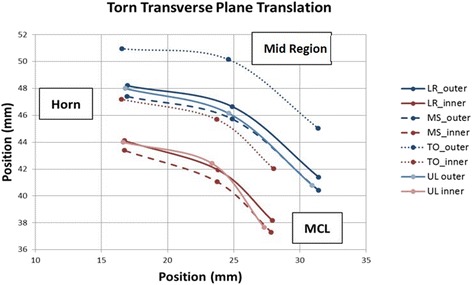
Torn Meniscus Graphic showing the combined translations and relative positions of the beads (deformations) in the transverse plane. Increased posterior translation is seen for the torn state

**Fig. 7:**
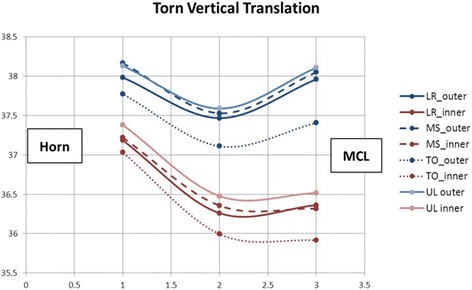
Torn Meniscus Graphic showing the combined translations and relative positions of the beads (deformations) in the vertical plane. Increased posterior translation is seen for the torn state

In the torn state, the displacement of the meniscus increased, and a different deformation pattern was observed compared to the intact state. During early stance phase, the meniscus had similar bulk translations and internal deformations, but when transitioning from mid-stance to toe-off, the torn meniscus had significantly greater posterior translation across the tibia than did the intact (3.44 ± 0.17 mm versus 1.67 ± 0.11 mm, *p* = 0.001). Significant flattening of the posterior meniscus was also observed during this movement compared to the intact state (0.4 ± 0.06 mm versus 0.2 ± 0.06 mm, *p* = 0.0056). Although not significant, intra-meniscal contractile deformations of the posterior horn (−0.4 ± 0.1 mm versus −0.2 ± 0.08 mm, *p* = 0.0909) and MCL regions (−0.21 ± 0.12 mm versus −0.11 ± 0.12 mm, *p* = 0.2264) tended to increase. The combined translation and deformation patterns for the torn state are shown graphically in Figs. 6 and 7.

**Fig. 8:**
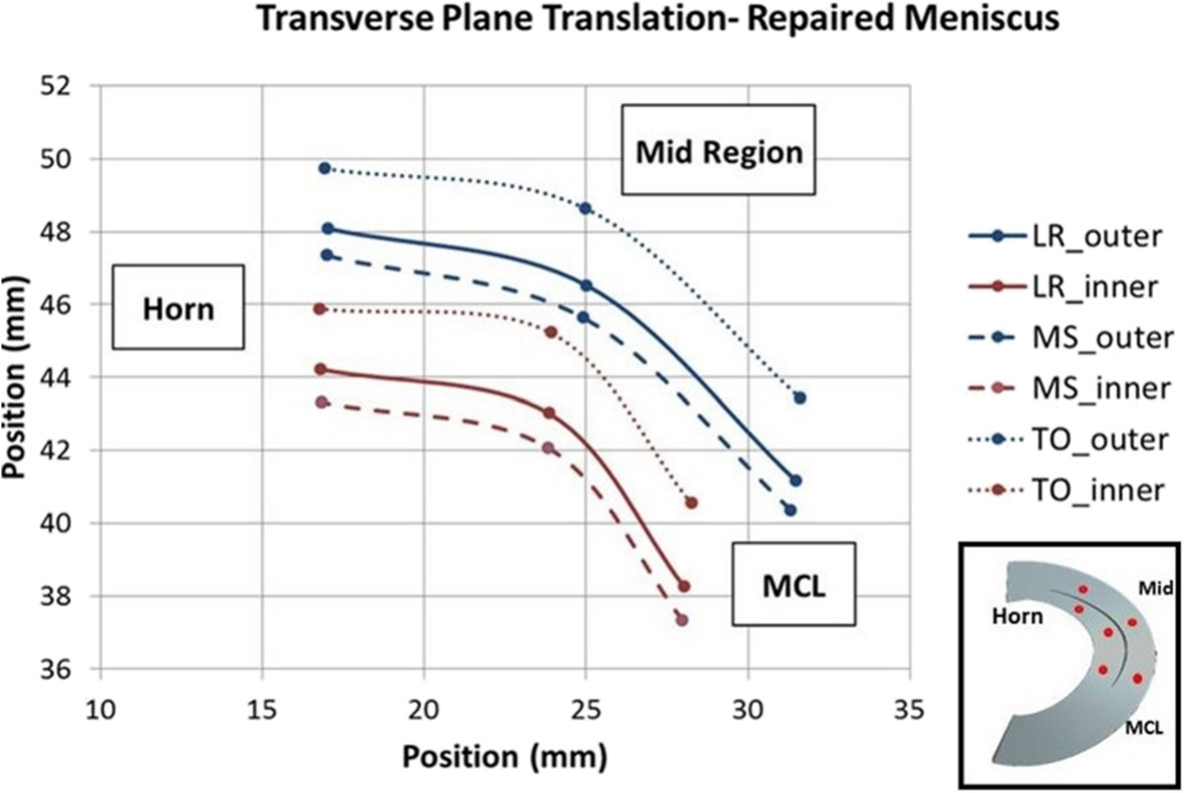
Repaired Meniscus Graphic showing the combined translations and relative positions of the beads (deformations) in the transverse plane. Excess posterior translation is improved in the repaired state

**Fig. 9:**
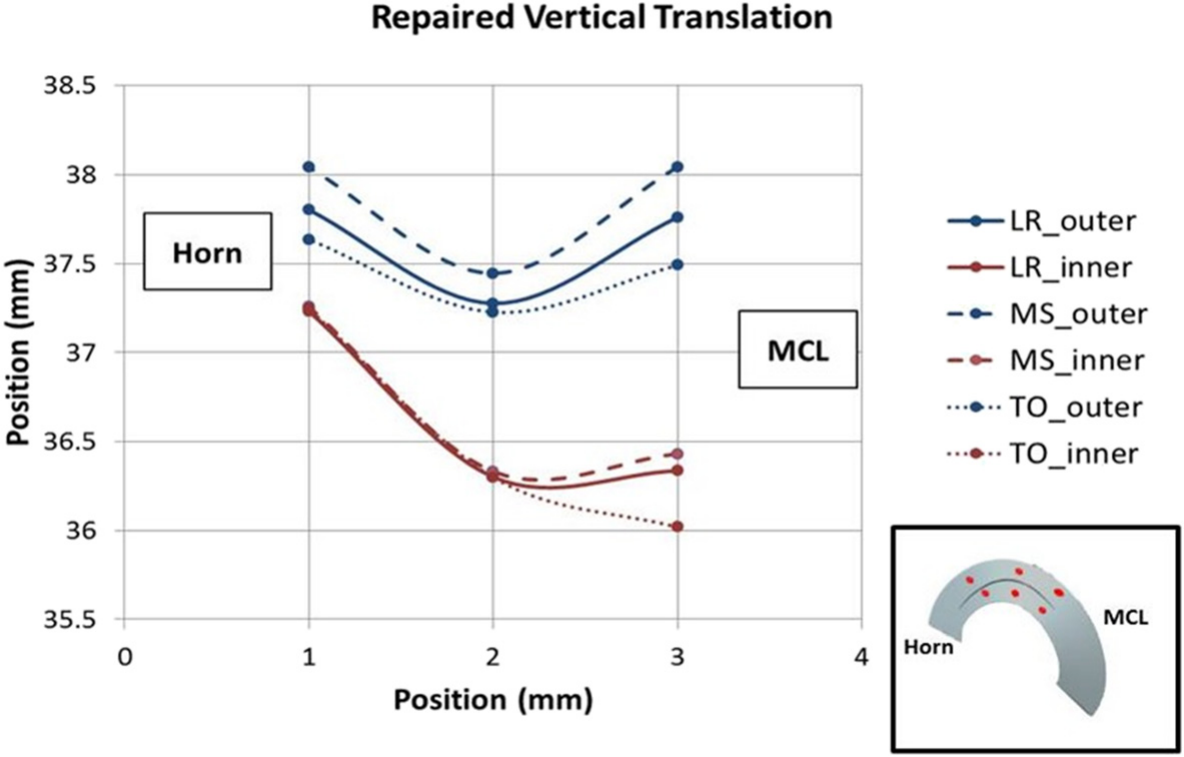
Repaired Meniscus Graphic showing the combined translations and relative positions of the beads (deformations) in the vertical plane. Excess posterior translation is improved in the repaired state

In the repaired state, the natural kinematics and deformations of the medial meniscus were improved compared to the torn state but only partially restored to the intact state. After repair, anterior displacement from loading response to mid stance increased slightly by 0.11 mm (possibly due to the tethering of the suture). However, the posterior displacement of the meniscus decreased from 3.44 ± 0.29 mm to 2.89 ± 0.32 mm during the transition from mid stance to toe-off. When compared to the respective intact values (1.67 ± 0.24 mm), there was still a significant difference between these two states (*p* = 0.0001). Table 2 shows that the integrity of the meniscus was restored in the SI direction (−0.27 ± 0.22 mm versus −0.2 ± 0.14 mm) as well as the ML direction (0.11 ± 0.15 mm versus 0.02 ± 0.13 mm), with no significant difference between the two states (*p* = 0.309, *p* = 0.3419). Internal meniscal deformations of the repaired state also closely resembled the natural meniscus during this movement. The combined translation and deformation patterns for the repaired state are shown graphically in Figs. 8 and 9.

Compression, not gapping, was seen between the bead pairs across the repaired meniscus. The 3D marker couple separation distances for the posterior horn, mid-body, and junctional regions were −0.2 ± 0.06 mm, −0.07 ± 0.17 mm, and −0.17 ± 0.09 mm, while the intact values were −0.2 ± 0.08 mm, −0.07 ± 0.06 mm, and −0.11 ± 0.12 mm respectively, with no significant differences between the two states (*p* = 0.98, *p* = 0.97, *p* = 0.76).

## Discussion

The kinematic effects of walking on either a meniscal tear or following meniscal repair have not been clearly understood. This has led to differences of opinion regarding the ideal regimen for knee rehabilitation following meniscal repair. This is the first study to report the acute response of a torn medial meniscus to functional loading before and after surgical repair. We hypothesized that meniscal repair would prevent gapping of meniscal edges in the presence of a longitudinal tear. Our data demonstrate that during the toe-off phase of the gait cycle, the edges of a longitudinal tear are loaded in compression, albeit with evidence of microscopic vertical separation. The posterior displacement of the meniscus with knee flexion is also dramatically increased in the presence of a longitudinal tear and is reduced once a suture repair has been performed. We also hypothesized that meniscal repair would restore the mechanical response of the intact meniscus during stance phase to that seen in the native meniscus. We found that the native meniscus experienced posterior translation, inferior displacement, and internal elongation while the knee was loaded. It internally rotated and progressively flexed during stance phase, which is similar to previous publications (Li et al. 2014; Morgan and Casscells 1986; Pujol et al. 2008; Sakai et al. 1996; Taylor et al. 2004). The meniscal tear produced greater posterior translation and inferior compression than the intact, which was improved with meniscal repair but not restored to normal.

The first objective of this study was to analyze the displacements and deformations of the intact meniscus during the stance phase. The native medial meniscus proved to be a dynamic structure under load as initial heel strike produced posterior translation and superior displacement of the meniscus. As flexion was decreased from heel strike to mid-stance, there was a reversal of both translation and internal deformation, with anterior translation and inferior compression of the meniscus. The transition from mid-stance to toe-off produced the greatest angle of knee flexion and the highest joint load during the loading cycle, and the meniscus responded with increased posterior translation to maintain congruence with the femoral condyle. In all phases of gait the least amount of meniscal displacement was observed at the posterior horn and the greatest was seen medially, adjacent to the MCL. There was also inferior displacement and medio-lateral elongation of the meniscus, which caused the beads placed in the inner and outer meniscal to move closer together with loading. This internal compression experienced by the native meniscus at increased flexion and load may play an important role in load distribution and shock absorption.

The second objective was to evaluate how displacement and deformation of the meniscus changes in the presence of a longitudinal tear. Our findings show that posterior translation of the meniscus was greater with flexion in the presence of a longitudinal tear (*p* = 0.0001) and that in this state there was an increased compression of the meniscal edges not observed in the native meniscus. The presence of a tear also led to lateral displacement of the meniscus under load, in contrast to the intact state, in which the meniscus translated purely posteriorly with increased knee flexion (*p* = 0.05). Furthermore, under physiologic loading, the torn meniscus underwent elongation and compression of the edges of the tear at toe-off, with slight vertical separation posteriorly. This signifies that the tear edges were moving independently and were likely unstable.

Lastly, we sought to determine the extent to which a suture repair restores the mechanical response of the meniscus to gait loading. MRI studies have shown that the posterior portion of the medial meniscus translates posterior with increasing flexion, the least excursion occurring at the posterior horn (Vedi et al. 1999). This phenomenon was seen in our experiments, where the native meniscus was displaced by an average of 1.67 mm posteriorly during toe-off compared to 3.43 mm in the torn state. Meniscal repair was shown to produce a statistically significant reduction in posterior translation (2.89 mm, *p* = 0.03) and improvement in mechanical response, though normal was not restored. Conversely, vertical load bearing displacement of the meniscus was more dynamic. Loading response caused superior displacement of the meniscus, suggesting that the initial load was borne anterior to the location of our implanted beads. At toe-off, the inferior component of meniscal displacement averaged 0.2 mm in the intact specimens, and there was a significant increase of inferior displacement in the torn state (*p* = 0.005). Suture repair was shown to restore the mechanical response to vertical load nearly identical to the intact state 0.27 mm, which approached statistical significance (*p* = 0.07). Regarding deformation, the repaired meniscus proved to be less dynamic than the intact state, as there was no superior bulging that occurred across meniscus fibers in the toe-off phase. There was radial and vertical internal fiber compression that occurred across the meniscus edges, preventing the tear edge gapping that was observed in the torn state, however, this did not reach statistical significance (*p* = 0.84).

Historically, postoperative immobilization of the knee in extension was thought to protect meniscus repairs from edge separation that could occur during flexion (Dowdy et al. 1995; Stärke et al. 2009; Taylor et al. 2004). A recent biomechanical analysis of longitudinal meniscus tears showed that meniscal compressive loads, not distractive loads, occur throughout full range of knee flexion and extension (Richards et al. 2005). Furthermore, the absence of distractive loads suggests that anatomic meniscal edge reduction may be more important than the ultimate strength of repair constructs (Rankin et al. 2002; Richards et al. 2005). Our results echo these findings as the entire intact, torn, and repaired menisci compressed and translated posteriorly with progressive flexion. Ganley et al. showed that minimal edge separation occurred in repaired and unrepaired longitudinal posterior meniscus tears when loaded with 45 kg at 60° of flexion (Ganley et al. 2000). As significant deformation of the menisci was not observed under these loading conditions, the authors concluded that partial weight bearing is acceptable after repair. In our protocol we applied much higher forces (161 kg of axial load and 18 kg of shear force) at 32° of flexion to simulate loading of the knee at toe-off, which more closely replicated the type of loading seen during walking. Again, we found no evidence of repair edge separation or suture failure (Bylski-Austrow et al. 1994; Ganley et al. 2000). Suture repair did cause visual evidence of decreased meniscal width, which is an important factor for healing. Pujol et al. showed a 9 % reduction in width on follow up CT arthrogram of completely healed repaired medial menisci (Pujol et al. 2008). They noted that meniscal repair reduces the tear to a narrower and more stable configuration and that a significant correlation exists between the rate of narrowing and healing, as their best clinical outcomes were obtained with narrowed, healed menisci.

The repair demonstrated micromotion at early phases of stance, but no gapping under the conditions examined. This micromotion was less than 0.2 mm at all regions, which is consistent in current literature, following vertical mattress repair (Muriuki et al. 2011; Rankin et al. 2002). Greater than 1 mm tear diastasis on MRI is predictive of re-tear, but the amount of repair site displacement necessary to adversely affect meniscal healing is unknown (Mariani et al. 1996). Functional stress with micromotion may provide some benefit to meniscus healing and strength through collagen remodeling, while prolonged immobilization may have deleterious effects on knee motion, muscle strength, ligaments and menisci (Barber 1994; Klein et al. 1982; Lin et al. 2013; Mariani et al. 1996; Shelbourne and Nitz 1990). Dowdy et al. showed more uniform collagen and a greater collagen content at 10 weeks after medial meniscal repair in fully mobilized dogs versus immobilized (Dowdy et al. 1995). They concluded that prolonged immobilization decreases collagen formation and has a detrimental effect on healing. Accelerated rehab protocols after meniscus repair have shown clinical success (Barber 1994; Klein et al. 1982; Mariani et al. 1996; Schimmer et al. 1998; Stein et al. 2010). Barber showed no statistical significance in failure rates between a protective repair protocol versus accelerated and concluded that activity restriction is not necessary after meniscal repair (Barber 1994). Likewise, O’Shea and Shelbourne reported a 55 % complete meniscal healing rate and 34 % partial healing rate in chronic bucket handle tears with ACL reconstruction and accelerated rehab, suggesting that early load does not prohibit healing (O'Shea and Shelbourne 2003).

In this study we chose to model acute longitudinal tears of the posterior medial meniscus as lesions of this type are frequently seen in traumatic athletic injuries and chronic ACL deficiency. However, we have no evidence to support extrapolation of our findings to other tear morphologies observed in the knee (Yoo et al. 2009). We measured meniscal displacement and internal deformation under static loading simulating the principal phases of weight-bearing during gait, and observed no suture failures. However, during rehabilitation after meniscal injury, mechanical failure of the suture construct may occur due to the cyclic loading conditions imposed on the knee joint (Komistek et al. 1998; Pujol et al. 2008; Taylor et al. 2004). It would be interesting from a clinical standpoint to add additional gait cycles which may better simulate the initial response in the weeks after surgery but prior to healing. However, this model which uses multiples of body weight would likely fail as cadaveric tissue begins to degrade. While we used multiples of bodyweight necessary to simulate walking, joint reactive forces across the knee with running and stair-climbing were not addressed by this study and significantly exceed those generated during walking. The order of testing was always intact first, as this was followed by tear creation. Ideally, one would vary the order of testing for the torn and repaired states to minimize the potential laxity of cadaveric soft tissues, which can occur during repeated testing. However, the torn state was tested after the repaired state by removing the sutures and minimizing the chance that a bead was lost, as a pilot study had shown the torn state to be the most mobile.

## Conclusion

These findings show that although the repaired meniscus is less dynamic than the intact, suture repair helps restore the loading response of the tissue and prevents harmful separation of the edges of the tear during stance phase. Moreover, meniscal repair generates tear narrowing and compression that is accentuated during flexion, which is a significant component of healing. These results support the clinical studies demonstrating that in a rehabilitation setting weight bearing protocols including normal walking may be initiated early in the postoperative period after repair of a longitudinal meniscal tear. Considering that meniscal repairs are often performed concomitantly with other procedures, such as ACL reconstruction, the knowledge that these repairs are not adversely affected by walking may be beneficial to patients during rehabilitation.
